# Comparison of real-world kidney outcomes with predicted outcomes at the time of diagnosis and post-biopsy in a cohort of children with IgA nephropathy

**DOI:** 10.1590/2175-8239-JBN-2024-0260en

**Published:** 2025-09-08

**Authors:** Mafalda Félix Cabral, Inês Martins, Miguel Pereira, Gonçalo Vale, Madalena Almeida Borges, Maria Soto-Maior Costa, Fernando Caeiro, Mário Góis, Helena Sousa, Telma Francisco, Gisela Neto, Margarida Abranches, Rute Baeta Baptista

**Affiliations:** 1 Hospital Dona Estefânia, Unidade Local de Saúde São José, Área de Pediatria, Lisboa, Portugal.; 2 Hospital Dona Estefânia, Unidade Local de Saúde São José, Unidade de Nefrologia Pediátrica, Lisboa, Portugal.; 3 Hospital Dona Estefânia, Unidade Local de Saúde São José, Unidade de Cuidados Intensivos Pediátricos, Lisboa, Portugal.; 4 Hospital Curry Cabral, Unidade Local de Saúde São José, Unidade de Nefrologia, Lisboa, Portugal.

**Keywords:** Child, Glomerulonephritis, IGA, International IgAN Prediction Tool, Primary Glomerulonephritis

## Abstract

**Introduction:**

IgA nephropathy (IgAN) is the most common form of primary glomerulonephritis, with a variable clinical course. This study aimed to evaluate real-world kidney outcomes in children with IgAN and compare these with predictions from the International IgAN Prediction Tool for children (IIgAN-PT).

**Methods:**

A single-center, longitudinal retrospective study was conducted on pediatric patients diagnosed with IgAN from 2010 to 2022. Data on clinical, laboratory, and histological parameters were analyzed. The IIgAN-PT score was calculated for each patient at biopsy and one year after biopsy. The primary outcome was a composite endpoint of ≥30% eGFR decrease or progression to end stage kidney disease (ESKD).

**Results:**

Among 23 patients (57% male, median age at biopsy 13.8 years), MEST-C scores showed M1 in 87%, E1 in 22%, S1 in 39%, T1/2 in 13%, and C1 in 26%. During a median 3.1-year follow-up, 26% reached the primary outcome, while the median predicted risk based on IIgAN-PT was 6.5%. Additionally, 57% experienced eGFR decline (annual median decline of 5.6 mL/min/1.73 m^2^). Application of the updated IIgAN-PT one-year post-biopsy (n = 13) resulted in a median predicted risk of 1.79%, while 23% met the primary outcome.

**Conclusion:**

The observed eGFR decline or progression to ESKD was higher than predicted, highlighting the need for early diagnosis, monitoring, and treatment. Small-scale studies like ours underscore the importance of early intervention and may inform the design of larger studies to improve the predictive ability of the IIgAN-PT tool in diverse clinical settings.

## Introduction

IgA nephropathy (IgAN) is the most common form of primary glomerulonephritis and presents with a highly variable clinical course and prognosis^1,2^. Among pediatric patients, approximately 20% progress to end-stage chronic kidney disease (ESKD) within 20 years of diagnosis^3^, while in adults, about 30% reach ESKD 10-20 years after diagnosis^4,5^.

In 2019, a tool was proposed to predict the risk of 50% decline in kidney function or progression to ESKD in patients with biopsy-proven IgAN^6^. Later, in 2021, there was an adaptation of the International IgA Nephropathy Prediction Tool (IIgAN-PT) for use in children. The original tool demonstrated poor agreement between predicted and observed risks, likely due to differing disease trajectories in children^7^. The pediatric IIgAN-PT comprises two models: with and without ethnicity. It is the first method to predict the risk of a 30% decline in estimated glomerular filtration rate (eGFR) or kidney failure in children at the time of biopsy using clinical risk factors and Oxford MEST-C histology scores^7^. In 2024, it was also proposed an application of the updated IIgAN-PT in children one or two years post-biopsy^8^.

We aimed to compare real-world kidney outcomes in a cohort of children with IgAN with the predicted kidney outcomes at the time of kidney biopsy, based on the adapted IIgAN-PT for children. We also aimed to compare real-world kidney outcomes in this cohort, re-evaluating the risk after biopsy with the application of the updated pediatric IIgAN-PT in children one-year post-biopsy.

## Methods

We conducted a single-center longitudinal retrospective study of patients aged 0 to under 18 years with a diagnosis of IgAN between 2010 and 2022. Patients with IgA vasculitis with nephritis and secondary IgAN were excluded from the analysis.

Demographic, clinical, laboratory, histological, treatment regimen, and complications data were obtained from medical charts in the hospital information system and analyzed. Diagnosis was proven by renal biopsy. The IIgAN-PT score was calculated for each patient according to their follow-up duration in the study.

A subgroup analysis was also performed. Two groups were defined: one that received corticosteroids prior to biopsy and another that did not undergo immunosuppressive therapy before the biopsy. The data was also analyzed according to age groups of below 12 years of age and 12 years or older. We also applied the updated IIgAN-PT retrospectively in children one or two-years post-biopsy, with a re-evaluation performed one year after biopsy. For this analysis, exclusion criteria were absence of any data necessary for score calculation one year post-biopsy and a follow-up duration of less than 12 months post-biopsy.

The variables comprised in the IIgAN-PT for use in pediatric patients at biopsy that were used to calculate the score were: age at biopsy, sex, height at biopsy, weight at biopsy, proteinuria based on a 24-hour collection or urine protein to creatinine ratio if a 24-hour collection was not available, serum creatinine at biopsy, systolic blood pressure at biopsy, diastolic blood pressure at biopsy, race, use of ACE inhibitor or ARB at the time of biopsy, MEST M-score, E-score, S-score, and T-score, and use of immunosuppression drug at or prior to biopsy.

The variables comprised in the updated IIgAN-PT for children one or two years post-biopsy that were used to calculate the score were: age at landmark time post-biopsy, sex, height at landmark time post-biopsy, weight at landmark time post-biopsy, proteinuria based on a 24-hour collection or urine protein to creatinine ratio if a 24-hour collection was not available at landmark time post-biopsy, serum creatinine at landmark time post-biopsy, systolic blood pressure at landmark time post-biopsy, diastolic blood pressure at landmark time post-biopsy, race, use of ACE inhibitor or ARB at landmark time post-biopsy, MEST M-score, MEST E-score, MEST S-score, MEST T-score, and use of immunosuppression drug at or prior to the landmark time post-biopsy. The risk of renal progression was determined 12–72 months after the landmark time post-biopsy, calculated for each patient according to their follow-up.

The primary outcome was defined as a composite endpoint of a ≥30% decrease in eGFR or progression to end stage kidney disease (ESKD). Secondary outcomes included the new onset of albuminuria/proteinuria and any reduction in eGFR from baseline.

Qualitative variables were expressed as percentages, while quantitative variables were reported as medians and interquartile ranges (IQR). Continuous variables were compared using the Mann-Whitney U test and categorial variables with Fisher’s exact test. STATA Statistical software for data science version 14 (^©^ Copyright 1996–2025 StataCorp LLC) was used to perform data analysis.

All data collected were obtained as part of routine clinical care, and no samples were collected specifically for this study. Data were collected and retained in accordance with the General Regulation on Data Protection (EU) 2016/679 of 27 April 2016. The study was submitted for approval to the local Ethics Committee.

## Results

We identified 23 patients, of whom 13 (57%) were male, with a median age at biopsy of 13.8 years (IQR 9.6–16.3). Regarding comorbidities, three patients (13%) were overweight, one (4%) was obese, three (13%) had asthma, one (4%) had dyslipidemia, and one (4%) had fibrous dysplasia. The median initial eGFR was 130.4 mL/min/1.73 m^2^ (IQR 114.6–150.1). The median proteinuria at the time of biopsy was 0.261 g/day/1.73 m^2^.

Five patients (22%) were receiving corticosteroid treatment prior to biopsy. MEST-C scores were as follows: M1 in 20 patients (87%), E1 in five patients (22%), S1 in nine patients (39%), T1/2 in three patients (13%), and C1 in six patients (26%).

Based on the IIgAN-PT score at biopsy, the median predicted risk of a ≥30% decline in eGFR or progression to ESKD, adjusted for each patient follow-up time in the study, was 6.5% (IQR 4.5–8.6) (Table 1).

**Table 1:** Demographic, clinical, laboratory, and histological variables at the time of biopsy

Variables		N = 23
Male, n (%)		13 (57)
Median age, years (IQR)		13.8 (9.6–16.3)
Median initial eGFR, mL/min/ 1.73 m^2^ (IQR)		130.4 (114.6–150.1)
Corticosteroids before, n (%)		5 (22)
MEST-C Score	M1, n (%)	20 (87)
	E1, n (%)	5 (22)
	S1, n (%)	9 (39)
	T1/2, n (%)	3 (13)
	C1, n (%)	6 (26)
Median predicted risk ≥30% decline in eGFR/ progression to ESKD, % (IQR)		6.5 (4.5–8.6)

During a median follow-up period of 3.1 years (IQR 1.7–7.3), six patients (26%) met the primary outcome, including one patient (4%) who underwent a kidney transplant. For secondary outcomes, two patients (9%) developed *de novo* albuminuria/proteinuria and 13 patients (57%) experienced a decline in eGFR.

Over the follow-up period, nine patients (39%) were treated with corticosteroids, and four received (17%) other immunosuppressants, including azathioprine, cyclophosphamide, and mycophenolate mofetil. Additionally, 19 patients (83%) were treated with angiotensin-converting enzyme inhibitors (ACEi) or angiotensin II receptor blockers (ARBs). The median annual decline in eGFR was 5.6 mL/min/1.73 m^2^ (IQR 1.98-17.4). (Table 2).

**Table 2:** Clinical, laboratory, and histological variables at the time of follow-up

Variables	N = 23
Median follow-up, years (IQR)	3.1 (1.7–7.3)
Corticosteroids, n (%)	9 (39.1)
Other immunosuppressors, n (%)	4 (17.4)
ACEi/ARBs, n (%)	19 (82.6)
Composite of ≥30% decrease in eGFR/ progression to ESKD, n (%)	6 (26.1)
Need for transplant, n (%)	1 (4.3)
New onset albuminuria/ proteinuria, n (%)	2 (9)
eGFR decrease, n (%)	13 (57)
Median annual decline in eGFR, mL/min/1.73 m^2^ (IQR)	–5.6 (–1.98; –17.4)

Regarding subgroup analysis, patients who underwent corticosteroid therapy prior to biopsy (n = 5) had a median age of 15.8 years. Among these, 40% (n = 2) reached the composite outcome and 60% later required additional immunosuppressive therapy. In the second group (n = 18), with a median age of 13.5 years, 22.2% reached the composite outcome and 6% required further immunosuppressive therapy.

In the subgroup of patients aged <12 years (n = 9), 33.3% (n = 3) reached the composite outcome. Six patients experienced a reduction in their eGFR, with a median annual decline in eGFR of 3.5 mL/min/1.73 m^2^ (IQR 2.56; 6.62). In the subgroup of patients aged ≥12 years (n = 14), 21.4% (n = 3) reached the composite outcome. Six patients in this group also experienced a reduction in eGFR, with a median annual decline in eGFR of 21.87 mL/min/1.73 m^2^ (IQR 4.00; 36.45).

After applying the defined criteria for reevaluation using the tool, 13 patients were analyzed, with a median age of 13.75 years (IQR 11.86–15.30), of whom 54% were male. The baseline eGFR was 149.42 mL/min/1.73 m^2^ (IQR 124.78–163.04). The median proteinuria at one-year post-biopsy was 0.13 g/day/1.73 m^2^.

Based on the updated IIgAN-PT for children applied post-biopsy, at the one-year landmark, the mean predicted risk of experiencing either a ≥30% decline in eGFR or progression to end-stage kidney disease (ESKD) adjusted for each patient’s follow-up time was 1.79% (IQR 1.19–2.36). Among the 13 patients, 23% (n = 3) reached the primary outcome (Table 3), who did not have the highest predicted risk according to the tool. Their predicted risks, adjusted for follow-up time, were 1.43, 2.09, and 2.29%. Conversely, three other patients with higher predicted risks (2.49, 3.47, and 7.79%) did not meet the composite outcome.

**Table 3:** Demographic, clinical, and laboratory variables 1 year post-biopsy and follow-up outcomes

Variables	N = 13
Male, n (%)	7 (54)
Median age, years (IQR)	13.75 (11.86; 15.30)
Median eGFR, mL/min/1.73 m^2^ (IQR)	149.42 (124.78; 163.04)
Median predicted risk ≥30% decline in eGFR/progression to ESKD, % (IQR)	1.79 (1.19–2.36)
Composite of ≥30% decrease in eGFR/progression to ESKD, n (%)	3 (23)

## Discussion

In our cohort, a decrease in eGFR ≥30% or progression to ESKD occurred at a rate four times higher than that predicted by the IIgAN-PT at the time of biopsy. When applying the tool 1 year post-biopsy, although to a smaller number of patients, the results were even more discrepant, with a decrease in eGFR ≥30% or progression to ESKD 13 times higher than that predicted by the IIgAN-PT at 1-year post-biopsy. Delayed diagnosis and referral from other centers may have contributed to advanced disease stages at biopsy and delayed initiation of immunosuppressive therapy, underscoring the importance of early and standardized diagnostic pathways for pediatric IgAN. Additionally, this cohort spans 14 years, a period during which clinical practices have evolved. Historically, renal biopsies for pediatric IgAN were often performed at more advanced stages of disease progression, marked by significant renal function loss, hypertension, or persistent proteinuria despite optimal renin-angiotensin system blockade. The prior conservative approach stemmed from the limited understanding of disease progression and insufficient data on early intervention. Presently, kidney biopsies are performed earlier in the disease course, supported by evidence suggesting that early intervention may delay or prevent progression to severe stages^9^. This shift has been influenced by new treatment options and a clearer understanding of risk factors for IgAN progression in children^10^.

Moreover, compared to the study of Cambier et al.^11^, which demonstrated that IgAN in children with minimal proteinuria at time of biopsy may be associated with both acute and chronic glomerular lesions, our patients exhibited a higher prevalence of chronic lesions. Specifically, our cohort showed a higher rate of mesangial proliferation (M1: 87.0 vs. 53.5%), extracapillary proliferation (C1: 26.0 vs. 7.1%), and tubular atrophy/interstitial fibrosis (T1/2: 13.0 vs. 7.1%).

The decline in eGFR observed in our cohort can be partially attributed to a delayed IgAN diagnosis and a higher eGFR at the time of biopsy. The predictive tool used for children differs from the one used for adult patients in terms of risk prediction timeframes and variables considered^6,7,12^. In children, eGFR changes over time follow a nonlinear pattern, with an increase until age 18, followed by a linear decline similar to what is observed in adults. A smaller initial increase and faster subsequent decline in eGFR are associated with a higher risk of progression, suggesting that children at a greater risk of a 30% decrease in eGFR are more likely to experience a 50% decline in eGFR as they reach adulthood^7^.

The pediatric predictive tool, however, was developed using risk factors derived primarily from adult data. A validation study conducted in a Chinese population suggests that the models did not effectively distinguish low- and high-risk groups. This finding underscores differences between the validation and derivation cohorts regarding demographic, clinical, pathological, and treatment characteristics^13^. More recently, in the updated prediction tool for children to re-evaluate risk one or two-years post-biopsy, eGFR trajectory following the baseline period of one year after biopsy was found to be non-linear. Patients at higher predicted risk often started with a lower eGFR and experienced a more rapid decline over time^8^. In our cohort, the patients at a higher predicted risk did not show a decline in eGFR superior to 30% and did not reach ESKD.

The pediatric population with IgAN more frequently presents with gross hematuria and higher levels of proteinuria. In pediatric cases, active lesions, such as mesangial proliferation and endocapillary hypercellularity, are observed more often than chronic lesions, such as S1 and T1-2 lesions^14^. Similarly, children with proteinuria exceeding 1 g per day who are treated with corticosteroids have a higher probability of achieving complete remission of proteinuria compared to adult patients^14^. The higher corticosteroid sensitivity observed in pediatric IgAN patients may be explained by histological differences. In children, proteinuria is often associated with glomerular proliferation, while in adults it is more commonly linked to chronic lesions^15^. The difference in corticosteroid response seems to parallel distinct biopsy findings, supporting the importance of early biopsy and timely initiation of treatment in children.

In our cohort, the median proteinuria one year after biopsy decreased, probably due to the response to immunosuppression. Several clinical trials in the pediatric population have demonstrated that steroid and immunosuppressive therapy, in combination with ACEi/ARBs, can reduce acute kidney injury and improve long-term renal survival in patients with proteinuria without severe side effects^16,17,18^. This contrasts with findings in adults who tend to experience a higher incidence of side effects^19,20,21^.

This allows us to conlcude that, in many cases, biopsy provides better information than what unfolds in the clinical course and it should be done in an early stage. These patients require constant surveillance, and it is crucial to initiate disease-modifying therapies at pediatric age, when the disease often begins and when there is potential for intervention.

Concerning our study weaknesses, we can describe the small sample size from a single center and a relatively short follow-up period. However, the prediction tool can only be applied to a maximum of 72 months post-biopsy (6 years). Regarding its strong points, the study was innovative in applying the tool to real-life cases in a European population through a 14-year period. The study highlights discrepancies between predicted and observed outcomes, adding to the knowledge base on IgAN in children, and reinforces that an early intervention and risk monitoring is compelling and important for clinical practice.

Concerning the risk prediction in the pediatric population, small-scale reproducibility studies like ours can offer valuable insights to enhance the design of larger studies aimed at improving the prediction ability of this tool across diverse clinical settings. Moving forward, we plan to conduct a larger and prospective study reflecting the updated practices in pediatric IgAN in recent years.

## Data Availability

The dataset that supports the findings of this study is not publicly available.
