# Supplementary material for: Comparison of real-world kidney outcomes with predicted outcomes at the time of diagnosis and post-biopsy in a cohort of children with IgA nephropathy
Source: J Bras Nefrol. 2025 Sep 8;47(3):e20240260. doi: 10.1590/2175-8239-JBN-2024-0260en (PMC12448116; doi:10.1590/2175-8239-JBN-2024-0260en)
Supplement: Translation

# Translation: Comparação de desfechos renais de mundo real com desfechos previstos no momento do diagnóstico e pós-biópsia em uma coorte de crianças com nefropatia por IgA

**DOI:** 10.1590/2175-8239-JBN-2024-0260pt

**Authors:** Mafalda Félix Cabral, Inês Martins, Miguel Pereira, Gonçalo Vale, Madalena Almeida Borges, Maria Soto-Maior Costa, Fernando Caeiro, Mário Góis, Helena Sousa, Telma Francisco, Gisela Neto, Margarida Abranches, Rute Baeta Baptista

**Affiliations:** 1 Hospital Dona Estefânia, Unidade Local de Saúde São José, Área de Pediatria, Lisboa, Portugal.; 2 Hospital Dona Estefânia, Unidade Local de Saúde São José, Unidade de Nefrologia Pediátrica, Lisboa, Portugal.; 3 Hospital Dona Estefânia, Unidade Local de Saúde São José, Unidade de Cuidados Intensivos Pediátricos, Lisboa, Portugal.; 4 Hospital Curry Cabral, Unidade Local de Saúde São José, Unidade de Nefrologia, Lisboa, Portugal.

**Keywords:** Criança, Glomerulonefrite por IGA, International IgAN Prediction Tool, Glomerulonefrite Primária

## Abstract

**Introdução:**

Nefropatia por IgA (NIgA) é a forma mais comum de glomerulonefrite primária, apresentando curso clínico variável. Este estudo teve como objetivo avaliar desfechos renais de mundo real em crianças com NIgA e compará-los com previsões da *International IgAN Prediction Tool* (IIgAN-PT) para crianças.

**Métodos:**

Realizou-se estudo retrospectivo longitudinal de centro único em pacientes pediátricos diagnosticados com NIgA de 2010-2022. Foram analisados dados sobre parâmetros clínicos, laboratoriais e histológicos. O escore IIgAN-PT foi calculado para cada paciente na biópsia e um ano pós-biópsia. O desfecho primário foi um desfecho composto de redução ≥30% da TFGe ou progressão para doença renal em estágio terminal (DRET).

**Resultados:**

Entre 23 pacientes (57% do sexo masculino , idade mediana na biópsia 13,8 anos), os escores MEST-C demonstraram M1 em 87%, E1 em 22%, S1 em 39%, T1/2 em 13% e C1 em 26%. Durante acompanhamento mediano de 3,1 anos, 26% atingiram o desfecho primário, enquanto o risco mediano previsto com base no IIgAN-PT foi 6,5%. Além disso, 57% apresentaram declínio da TFGe (declínio anual mediano de 5,6 mL/min/1,73 m^2^). A aplicação do IIgAN-PT atualizado um ano pós-biópsia (n = 13) resultou em risco mediano previsto de 1,79%, enquanto 23% atingiram o desfecho primário.

**Conclusão:**

O declínio observado da TFGe ou progressão para DRET foi maior que o previsto, destacando a necessidade de diagnóstico, monitoramento e tratamento precoces. Estudos de pequena escala, como o nosso, ressaltam a importância da intervenção precoce e podem orientar o desenho de estudos maiores para aprimorar a capacidade preditiva da ferramenta IIgAN-PT em diversos cenários clínicos.

## Introdução

A nefropatia por IgA (NIgA) é a forma mais comum de glomerulonefrite primária e apresenta um curso clínico e prognóstico altamente variáveis^1,2^. Entre os pacientes pediátricos, aproximadamente 20% evoluem para doença renal crônica em estágio terminal (DRET) dentro de 20 anos após o diagnóstico^3^, enquanto em adultos, cerca de 30% atingem a DRET de 10 a 20 anos após o diagnóstico^4,5^.

Em 2019, foi proposta uma ferramenta para predizer o risco de declínio de 50% na função renal ou progressão para DRET em pacientes com NIgA comprovada por biópsia^6^. Posteriormente, em 2021, houve uma adaptação da *International IgA Nephropathy Prediction Tool* (IIgAN-PT) para uso em crianças. A ferramenta original demonstrou baixa concordância entre os riscos previstos e observados, provavelmente devido às diferentes trajetórias da doença em crianças^7^. O IIgAN-PT pediátrico consiste em dois modelos: com e sem a variável etnia. É o primeiro método a predizer o risco de um declínio de 30% na taxa de filtração glomerular estimada (TFGe) ou insuficiência renal em crianças no momento da biópsia utilizando fatores de risco clínicos e os escores histológicos Oxford MEST-C^7^. Em 2024, também foi proposta a aplicação da versão atualizada do IIgAN-PT em crianças um ou dois anos após a biópsia^8^.

Nosso objetivo foi comparar os desfechos renais de mundo real em uma coorte de crianças com NIgA com os desfechos renais previstos no momento da biópsia renal, com base no IIgAN-PT adaptado para crianças. Também buscamos comparar os desfechos renais de mundo real nessa coorte, reavaliando o risco após a biópsia com a aplicação da versão atualizada do IIgAN-PT pediátrico em crianças um ano pós-biópsia.

## Métodos

Realizamos um estudo retrospectivo longitudinal de centro único de pacientes com idade entre 0 e menos de 18 anos com diagnóstico de NIgA entre 2010 e 2022. Os pacientes com vasculite por IgA com nefrite e NIgA secundária foram excluídos da análise.

Com base nos prontuários médicos do sistema de informações do hospital, foram obtidos e analisados dados demográficos, clínicos, laboratoriais, histológicos, do regime de tratamento e das complicações. O diagnóstico foi comprovado por biópsia renal. O escore IIgAN-PT foi calculado para cada paciente de acordo com a duração do acompanhamento no estudo.

Também foi realizada uma análise de subgrupos. Dois grupos foram definidos: um que recebeu corticosteroides antes da biópsia e outro que não foi submetido à terapia imunossupressora previamente à biópsia. Os dados também foram analisados de acordo com as faixas etárias: menores de 12 anos de idade e 12 anos ou mais. Também aplicamos retrospectivamente o IIgAN-PT atualizado em crianças um ou dois anos pós-biópsia, com uma reavaliação realizada um ano após a biópsia. Para essa análise, os critérios de exclusão foram a ausência de quaisquer dados necessários para o cálculo do escore um ano após a biópsia e uma duração de acompanhamento inferior a 12 meses após a biópsia.

As variáveis incluídas no IIgAN-PT para uso em pacientes pediátricos no momento da biópsia, utilizadas para o cálculo do escore, foram: idade na biópsia, sexo, altura na biópsia, peso na biópsia, proteinúria com base em coleta de 24 horas ou relação proteína/creatinina urinária caso a coleta de 24 horas não estivesse disponível, creatinina sérica na biópsia, pressão arterial sistólica na biópsia, pressão arterial diastólica na biópsia, raça, uso de inibidor da ECA ou BRA no momento da biópsia, escore MEST M, escore E, escore S e escore T, e uso de imunossupressores na biópsia ou antes dela.

As variáveis incluídas na versão atualizada do IIgAN-PT para crianças um ou dois anos após a biópsia, utilizadas para calcular o escore, foram: idade no momento de referência pós-biópsia, sexo, altura no momento de referência pós-biópsia, peso no momento de referência pós-biópsia, proteinúria com base na coleta de 24 horas ou relação proteína/creatinina urinária caso a coleta de 24 horas não estivesse disponível no momento de referência pós-biópsia, creatinina sérica no momento de referência pós-biópsia, pressão arterial sistólica no momento de referência pós-biópsia, pressão arterial diastólica no momento de referência pós-biópsia, raça, uso de inibidor da ECA ou BRA no momento de referência pós-biópsia, escore MEST M, escore MEST E, escore MEST S, escore MEST T e uso de imunossupressores no momento de referência pós-biópsia ou antes dele. O risco de progressão renal foi determinado de 12 a 72 meses após o tempo de referência pós-biópsia, calculado para cada paciente de acordo com seu período de acompanhamento.

O desfecho primário foi definido como um desfecho composto de uma redução ≥30% na TFGe ou progressão para doença renal em estágio terminal (DRET). Os desfechos secundários incluíram albuminúria/proteinúria de início recente e qualquer redução na TFGe em relação ao valor basal.

As variáveis qualitativas foram expressas como porcentagens, enquanto as variáveis quantitativas foram reportadas como medianas e intervalos interquartis (IIQ). As variáveis contínuas foram comparadas por meio do teste U de Mann-Whitney e as variáveis categóricas com o teste exato de Fisher. Para a análise dos dados, foi utilizado o software *STATA Statistical for Data Science* versão 14 (© Copyright 1996–2025 StataCorp LLC).

Todos os dados coletados foram obtidos como parte do atendimento clínico de rotina e nenhuma amostra foi coletada especificamente para este estudo. Os dados foram coletados e armazenados em conformidade com o Regulamento Geral sobre Proteção de Dados (UE) 2016/679 de 27 de abril de 2016. O estudo foi submetido à aprovação do Comitê de Ética local.

## Resultados

Identificamos 23 pacientes, dos quais 13 (57%) eram do sexo masculino, com idade mediana na biópsia de 13,8 anos (IIQ 9,6–16,3). Com relação às comorbidades, três pacientes (13%) estavam acima do peso, um (4%) era obeso, três (13%) tinham asma, um (4%) tinha dislipidemia e um (4%) tinha displasia fibrosa. A mediana da TFGe inicial foi de 130,4 mL/min/1,73 m^2^ (IIQ 114,6–150,1). A mediana da proteinúria no momento da biópsia foi de 0,261 g/dia/1,73 m^2^.

Cinco pacientes (22%) estavam em tratamento com corticosteroides antes da biópsia. Os escores do MEST-C foram os seguintes: M1 em 20 pacientes (87%), E1 em cinco pacientes (22%), S1 em nove pacientes (39%), T1/2 em três pacientes (13%) e C1 em seis pacientes (26%).

Com base no escore IIgAN-PT na biópsia, o risco mediano previsto de uma redução de ≥30% na TFGe ou progressão para DRET, ajustado para o tempo de acompanhamento de cada paciente no estudo, foi de 6,5% (IIQ 4,5–8,6) (Tabela 1).

**Tabela 1:** Variáveis demográficas, clínicas, laboratoriais e histológicas no momento da biópsia

Variáveis		N = 23
Sexo masculino, n (%)		13 (57)
Idade mediana, anos (IIQ)		13,8 (9,6–16,3)
TFGe inicial mediana, mL/min/1,73 m^2^ (IIQ)		130,4 (114,6–150,1)
Corticosteroides prévios, n (%)		5 (22)
Escore MEST-C	M1, n (%)	20 (87)
	E1, n (%)	5 (22)
	S1, n (%)	9 (39)
	T1/2, n (%)	3 (13)
	C1, n (%)	6 (26)
Risco mediano previsto ≥30% de declínio na TFGe/ progressão para DRET, % (IIQ)		6,5 (4,5–8,6)

Durante um período mediano de acompanhamento de 3,1 anos (IIQ 1,7–7,3), seis pacientes (26%) atingiram o desfecho primário, incluindo um paciente (4%) que foi submetido à transplante renal. Quanto aos desfechos secundários, dois pacientes (9%) desenvolveram albuminúria/proteinúria *de novo* e 13 pacientes (57%) apresentaram declínio na TFGe.

Durante o período de acompanhamento, nove pacientes (39%) foram tratados com corticosteroides e quatro receberam (17%) outros imunossupressores, incluindo azatioprina, ciclofosfamida e micofenolato de mofetila. Além disso, 19 pacientes (83%) foram tratados com inibidores da enzima conversora de angiotensina (IECA) ou bloqueadores do receptor de angiotensina II (BRA). A mediana do declínio anual da TFGe foi de 5,6 mL/min/1,73 m^2^ (IIQ 1,98–17,4). (Tabela 2).

**Tabela 2:** Variáveis clínicas, laboratoriais e histológicas no momento do acompanhamento

Variáveis	N = 23
Mediana de acompanhamento, anos (IIQ)	3,1 (1,7–7,3)
Corticosteroides, n (%)	9 (39,1)
Outros imunossupressores, n (%)	4 (17,4)
IECA/BRA, n (%)	19 (82,6)
Desfecho composto de redução de ≥30% na TFGe/progressão para DRET, n (%)	6 (26,1)
Necessidade de transplante, n (%)	1 (4,3)
Albuminúria/proteinúria de início recente, n (%)	2 (9)
Redução da TFGe, n (%)	13 (57)
Declínio anual mediano da TFGe, mL/min/1,73 m^2^ (IIQ)	–5,6 (–1,98; –17,4)

Com relação à análise de subgrupos, os pacientes submetidos à terapia com corticosteroides antes da biópsia (n = 5) apresentaram uma mediana de idade de 15,8 anos. Entre eles, 40% (n = 2) alcançaram o desfecho composto e 60% necessitaram posteriormente de terapia imunossupressora adicional. No segundo grupo (n = 18), com idade mediana de 13,5 anos, 22,2% alcançaram o desfecho composto e 6% necessitaram de terapia imunossupressora adicional.

No subgrupo de pacientes com idade <12 anos (n = 9), 33,3% (n = 3) atingiram o desfecho composto. Seis pacientes apresentaram redução em sua TFGe, com um declínio anual mediano na TFGe de 3,5 mL/min/1,73 m^2^ (IIQ 2,56; 6,62). No subgrupo de pacientes com idade ≥12 anos (n = 14), 21,4% (n = 3) alcançaram o desfecho composto. Seis pacientes desse grupo também apresentaram uma redução na TFGe, com um declínio anual mediano de 21,87 mL/min/1,73 m^2^ (IIQ 4,00; 36,45).

Após a aplicação dos critérios definidos para reavaliação utilizando a ferramenta, 13 pacientes foram analisados, com idade mediana de 13,75 anos (IIQ 11,86–15,30), dos quais 54% eram do sexo masculino. A TFGe basal foi de 149,42 mL/min/1,73 m^2^ (IIQ 124,78–163,04). A mediana da proteinúria em um ano após a biópsia foi de 0,13 g/dia/1,73 m^2^.

Com base no IIgAN-PT atualizado para crianças aplicado pós-biópsia, no marco de um ano, o risco médio previsto de sofrer uma redução ≥30% na TFGe ou progressão para doença renal em estágio terminal (DRET) ajustado para o tempo de acompanhamento de cada paciente foi de 1,79% (IIQ 1,19–2,36). Entre os 13 pacientes, 23% (n = 3) atingiram o desfecho primário (Tabela 3), os quais não apresentaram o maior risco previsto de acordo com a ferramenta. Seus riscos previstos, ajustados para o tempo de acompanhamento, foram de 1,43, 2,09 e 2,29%. Por outro lado, três outros pacientes com maiores riscos previstos (2,49, 3,47 e 7,79%) não atingiram o desfecho composto.

**Tabela 3:** Variáveis demográficas, clínicas e laboratoriais 1 ano pós-biópsia e desfechos de acompanhamento

Variáveis	N = 13
Sexo masculino, n (%)	7 (54)
Idade mediana, anos (IIQ)	13,75 (11,86; 15,30)
TFGe mediana, mL/min/1,73 m^2^ (IIQ)	149,42 (124,78; 163,04)
Risco previsto mediano ≥30% de declínio na TFGe/progressão para DRET, % (IIQ)	1,79 (1,19–2,36)
Desfecho composto de redução de ≥30% na TFGe/progressão para DRET, n (%)	3 (23)

## Discussão

Em nossa coorte, uma redução na TFGe ≥30% ou a progressão para DRET ocorreram em uma taxa quatro vezes maior do que a prevista pelo IIgAN-PT no momento da biópsia. Ao aplicar a ferramenta um ano após a biópsia, embora a um número menor de pacientes, os resultados foram ainda mais discrepantes, com uma diminuição da TFGe ≥30% ou progressão para DRET 13 vezes maior do que a prevista pelo IIgAN-PT dentro do mesmo período. O diagnóstico tardio e o encaminhamento de outros centros podem ter contribuído para os estágios avançados da doença no momento da biópsia e para o início tardio da terapia imunossupressora, ressaltando a importância de vias de diagnóstico precoces e padronizadas para a NIgA pediátrica. Além disso, essa coorte abrange um período de 14 anos, durante o qual as práticas clínicas evoluíram. Historicamente, as biópsias renais para NIgA pediátrica eram frequentemente realizadas em estágios mais avançados da progressão da doença, marcados por perda significativa da função renal, hipertensão ou proteinúria persistente, apesar do bloqueio ideal do sistema renina-angiotensina. A abordagem conservadora adotada anteriormente era decorrente da compreensão limitada da progressão da doença e de dados insuficientes sobre a intervenção precoce. Atualmente, as biópsias renais são realizadas em estágios iniciais da doença, apoiadas por evidências que sugerem que a intervenção precoce pode atrasar ou evitar a progressão para estágios graves9. Essa mudança foi influenciada por novas opções de tratamento e por uma compreensão mais clara dos fatores de risco para a progressão da NIgA em crianças^10^.

Além disso, em comparação com o estudo de Cambier et al.^11^, que demonstrou que a NIgA em crianças com proteinúria mínima no momento da biópsia pode estar associada tanto a lesões glomerulares agudas quanto crônicas, nossos pacientes apresentaram maior prevalência de lesões crônicas. Especificamente, nossa coorte apresentou uma taxa mais elevada de proliferação mesangial (M1: 87,0 vs. 53,5%), proliferação extracapilar (C1: 26,0 vs. 7,1%) e atrofia tubular/fibrose intersticial (T1/2: 13,0 vs. 7,1%).

O declínio da TFGe observado em nossa coorte pode ser parcialmente atribuído a um diagnóstico tardio de NIgA e a uma TFGe mais alta no momento da biópsia. A ferramenta preditiva utilizada para crianças difere da utilizada para pacientes adultos em termos de prazos de predição de risco e variáveis consideradas^6,7,12^. Em crianças, as alterações da TFGe ao longo do tempo seguem um padrão não linear, com um aumento até os 18 anos, seguido de um declínio linear semelhante ao observado em adultos. Um aumento inicial menor e um declínio subsequente mais rápido na TFGe estão associados a um risco maior de progressão, sugerindo que crianças com risco mais elevado de uma redução de 30% na TFGe têm maior probabilidade de apresentar um declínio de 50% na TFGe ao atingirem a idade adulta^7^.

A ferramenta preditiva pediátrica, entretanto, foi desenvolvida utilizando fatores de risco derivados principalmente de dados de adultos. Um estudo de validação realizado em uma população chinesa sugere que os modelos não distinguiram com eficácia os grupos de baixo e alto risco. Esse achado ressalta as diferenças entre as coortes de validação e derivação em relação às características demográficas, clínicas, patológicas e de tratamento^13^. Mais recentemente, na ferramenta de predição atualizada para crianças, para reavaliar o risco um ou dois anos após a biópsia, constatou-se que a trajetória da TFGe seguinte ao período basal de um ano após a biópsia não era linear. Pacientes com risco previsto mais elevado frequentemente iniciaram com uma TFGe mais baixa e apresentaram um declínio mais rápido ao longo do tempo^8^. Em nossa coorte, os pacientes com maior risco previsto não apresentaram um declínio na TFGe superior a 30% e não atingiram a DRET.

A população pediátrica com NIgA apresenta com mais frequência hematúria macroscópica e níveis mais elevados de proteinúria. Em casos pediátricos, lesões ativas, como proliferação mesangial e hipercelularidade endocapilar, são observadas mais frequentemente do que lesões crônicas, como as lesões S1 e T1-214. De forma semelhante, crianças com proteinúria superior a 1 g por dia que são tratadas com corticosteroides têm uma probabilidade maior de obter remissão completa da proteinúria em comparação com pacientes adultos^14^. A maior sensibilidade aos corticosteroides observada em pacientes pediátricos com NIgA pode ser explicada por diferenças histológicas. Em crianças, a proteinúria é geralmente associada à proliferação glomerular, enquanto em adultos é mais comumente associada a lesões crônicas^15^. A diferença na resposta aos corticosteroides parece refletir os achados distintos da biópsia, sustentando a importância da biópsia precoce e do início oportuno do tratamento em crianças.

Em nossa coorte, a proteinúria mediana um ano após a biópsia diminuiu, provavelmente devido à resposta à imunossupressão. Diversos estudos clínicos na população pediátrica demonstraram que a terapia com esteroides e imunossupressores, em combinação com IECAs/BRAs, pode reduzir a injúria renal aguda e melhorar a sobrevida renal em longo prazo em pacientes com proteinúria sem efeitos adversos graves^16,17,18^. Isso contrasta com os achados em adultos que tendem a apresentar uma incidência maior de efeitos colaterais^19,20,21^.

Isso nos permite concluir que, em muitos casos, a biópsia fornece informações mais precisas do que as que se revelam no curso clínico, devendo ser realizada em um estágio inicial. Esses pacientes requerem vigilância constante e é fundamental iniciar terapias modificadoras da doença na idade pediátrica, fase em que a doença geralmente tem início e quando existe potencial para intervenção.

Quanto aos pontos fracos de nosso estudo, podemos descrever o pequeno tamanho da amostra proveniente de centro único e um período de acompanhamento relativamente curto. No entanto, a ferramenta de predição pode ser aplicada somente a um máximo de 72 meses após a biópsia (6 anos). Em relação aos seus pontos fortes, o estudo foi inovador ao aplicar a ferramenta a casos reais em uma população europeia no decorrer de um período de 14 anos. O estudo destaca discrepâncias entre os desfechos previstos e observados, contribuindo para a base de conhecimento sobre a NIgA em crianças, e reforça que a intervenção precoce e o monitoramento de riscos são convincentes e importantes para a prática clínica.

Em relação à predição de risco na população pediátrica, estudos de reprodutibilidade em pequena escala, como o nosso, podem oferecer *insights* valiosos para aprimorar o desenho de estudos maiores com o objetivo de melhorar a capacidade de predição dessa ferramenta em diversos ambientes clínicos. Para os próximos passos, planejamos realizar um estudo prospectivo e maior que reflita as práticas atualizadas em NIgA pediátrica nos últimos anos.

## Data Availability

O conjunto de dados que dá suporte aos resultados deste estudo não está disponível publicamente.
